# Trichinellosis-Induced Eosinophilic Myocarditis Mimicking Hypereosinophilic Syndrome

**DOI:** 10.7759/cureus.58946

**Published:** 2024-04-24

**Authors:** Manasawee Tanariyakul, Jonathan Estaris, Sakditad Saowapa

**Affiliations:** 1 Internal Medicine, University of Hawaii John A. Burns School of Medicine, Honolulu, USA; 2 Internal Medicine, Texas Tech University Health Sciences Center, Lubbock, USA

**Keywords:** bicuspid aortic valve disease, aortic stenosis (as), myocarditis, trichinella spiralis, hypereosinophilia, trichinellosis

## Abstract

*Trichinella spiralis*is an uncommon parasitic disease contracted through the consumption of undercooked pork. We report the case of a 59-year-old man with a history of bicuspid aortic valve with recent travel to the Philippines and consumption of raw pork presenting with progressive myalgia and hypereosinophilia (nadir 12,940/uL) in profound cardiogenic shock in the setting of critical aortic stenosis. He underwent emergent balloon valvuloplasty, which was complicated by aortic insufficiency. This necessitated a transcatheter aortic valve replacement. However, despite hemodynamic stabilization, he developed catastrophic eosinophilic myocarditis, complicated by cardiac arrest from ventricular tachycardia. A rectus femoris muscle biopsy confirmed the diagnosis, showing a *T. spiralis* parasite and significant eosinophilic infiltration. Empiric treatment with albendazole, ivermectin, and methylprednisolone resulted in the significant resolution of symptoms and the liberalization of critical illness. This case highlights the challenges of diagnosing the underlying etiologies of hypereosinophilia and/or eosinophilic myocarditis, underscoring the importance of considering parasitic etiologies, particularly in endemic regions or in patients who have a significant travel history to such areas. Prompt diagnosis and treatment are essential to prevent morbidity and mortality.

## Introduction

Trichinellosis is an uncommon parasitic disease that typically causes myopathy and gastrointestinal symptoms after the consumption of undercooked meat. The primary sources of infection are pork and its by-products [1]. Although it is usually a self-limiting illness, it has the potential to be lethal. Domestic and wild pigs are reservoirs of *Trichinella*, the intestinal roundworm responsible for trichinosis. Additional hosts for *Trichinella* include armadillos, dogs, cats, and brown rats, among other synanthropic creatures. However, wildlife can also serve as a reservoir, especially if the meat is improperly prepared or frozen. Since 1975, there has been an increasing association between trichinosis and the consumption of bear and walrus meat.

*Trichinella* has a widespread presence, ranging from the tropics to the Arctic, attributable to its high infectivity and the global distribution of pork. According to the CDC, approximately 10,000 cases of trichinellosis are reported globally every year [2]. Severe trichinellosis infection can affect multiple organs, including the central nervous, cardiac, and pulmonary systems. Although cardiac involvement in the disease is relatively rare compared to other organs, such as CNS involvement, which can be found in up to 24% of cases [3], it still occurs in 5-20% of moderate-severe infections and typically presents later in the disease course. The manifestation of cardiac involvement is less common and usually limited to pericardial effusions or nonspecific ECG abnormalities [4].

## Case presentation

**Figure 1:**
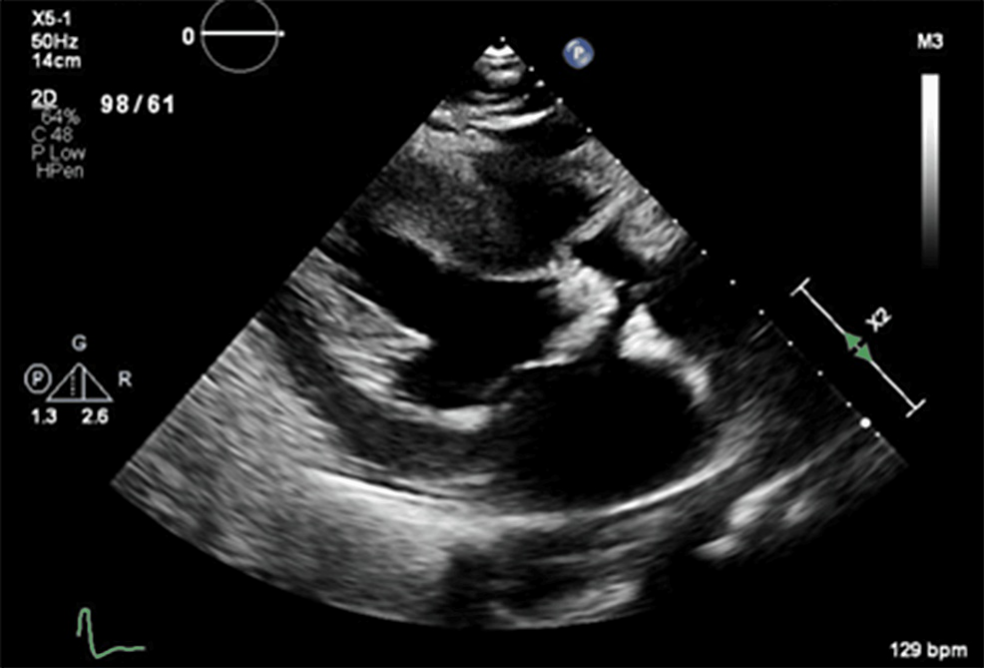
Parasternal long-axis view on transthoracic echocardiography demonstrating severe calcification of the aortic valve

**Figure 2:**
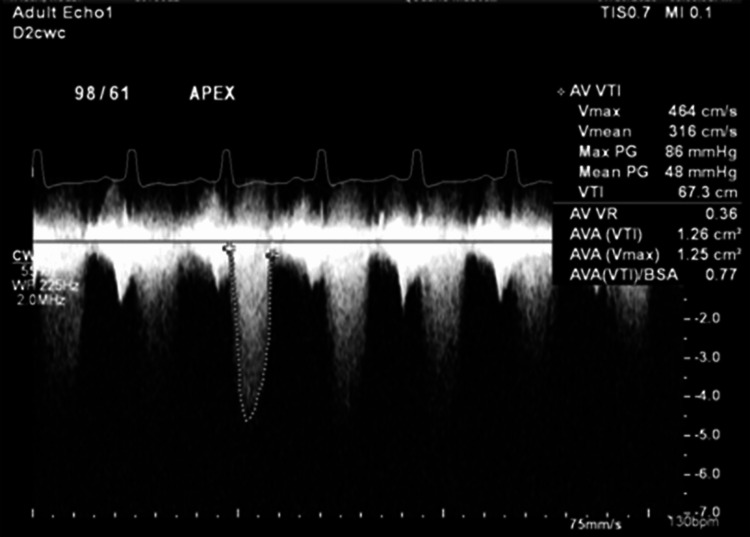
Continuous wave Doppler through the aortic valve, emphasizing a severely elevated mean pressure gradient and velocity

The patient was a 59-year-old male with a known history of bicuspid aortic valve without stenosis, routinely followed by a cardiologist prior to his travel to the Philippines, where he admitted to consumption of raw pork. In the months following his return, he experienced recurrent episodes of syncope, severe, progressively worsening symmetrical proximal myalgias, and weakness with associated pleuritic chest pain. He was not on any medications prior to admission. Upon presentation to the emergency department, he was in cardiogenic shock. An initial transthoracic echocardiography showed a left ventricular ejection fraction of 55-60%, normal right ventricular function, and new severe aortic stenosis with an aortic valvular area of 0.8 cm². The mean gradient across the aortic valve was 78 mmHg with a peak velocity of 5.6 m/s, and no aortic regurgitation was detected (Figure 1, 2). Due to hemodynamic instability in the setting of a new critical aortic stenosis requiring vasopressors, an emergent balloon valvuloplasty was performed, which improved the mean gradient from 87 to 35 mmHg. However, the patient developed severe aortic insufficiency and subsequently required transcatheter aortic valve replacement.

Despite initial stabilization of lactic acid, troponin levels and inflammatory markers such as C-reactive peptide continued to rise, along with persistent leukocytosis and hypereosinophilia with an absolute eosinophil of 12,940/uL (Table 1). Of note, a repeat echocardiogram showed the development of pericardial effusion with the symptomatic persistence of chest pain. Even following successful transcatheter aortic valve replacement, profound myocardial inflammation resulted in ventricular tachycardia, resulting in cardiac arrest with the return of spontaneous circulation. Given the clinical picture, there was high suspicion of eosinophilic myocarditis, eosinophilic polymyositis, and hypereosinophilic syndrome. The patient was empirically treated with high-dose pulse methylprednisolone. Cardiac MRI and endomyocardial biopsy were unsuccessful due to the patient’s inability to tolerate anesthesia and the procedure. A rectus femoris muscle biopsy found scattered foci of chronic endomysial and perivascular inflammation associated with focal increased eosinophils, along with scattered parasitic organisms morphologically consistent with *Trichinella spiralis*. Of note, he was also incidentally found to have Strongyloides IgG positivity. Further directed therapy with albendazole, ivermectin, and methylprednisolone resulted in the complete resolution of the critical illness. Therapy also included aspirin 81 mg daily indefinitely and clopidogrel 75 mg daily for three months for the new valvuloplasty.

**Table 1:** Patient’s initial laboratory results ALP, alkaline phosphatase; ALT, alanine aminotransferase; AST, aspartate aminotransferase; H, high; IV, index value; L, low; MCV, mean corpuscular volume

Parameter	Initial presentation	Normal range
Hemoglobin (g/dl)	11.1 (L)	11.2-15.7
Hematocrit (%)	34.0 (L)	34.1-44.9
MCV (fL)	81.3	79.4-98.4
WBC (×10^3^/uL)	35.06 (H)	3.80-10.80
Platelet (×10^3^/uL)	161	151-424
Absolute eosinophil count (×10^3^/uL)	12.94 (H)	0.04-0.54
BUN (mg/dL)	23	6-23
Creatinine (mg/dL)	0.8	0.6-1.4
AST (IU/L)	134 (H)	0-40
ALT (IU/L)	131 (H)	0-41
ALP (IU/L)	46	35-129
Total bilirubin (mg/dL)	0.3	0-1.2
Direct bilirubin (mg/dL)	<0.2	0-0.3
Troponin T Gen 5 (ng/L)	378 (then increases to 1,466 ng/L at peak) (H)	<19
Serum IgE (IU/mL)	722.0 (H)	<100
Aldolase (U/L)	75.7 (H)	<8.1
C-reactive protein (mg/L)	18.5 (H)	<10.0
Strongyloides IgG (IV)	2.3 (H)	<0.9

## Discussion

Trichinellosis is a parasitic disease transmitted through the consumption of infected meat, particularly pork, and is prevalent worldwide. The illness is prevalent in Latin America, Eastern Europe, and Asia [5]. Humans may become infected by eating undercooked meat from animals such as horses, pigs, and wild boars that are infected with *T. spiralis* larvae [4]. Human trichinellosis has been reported in 55 different nations worldwide, and it is becoming a significant public health issue. Asia has also had a significant number of trichinellosis outbreaks, mostly in China and Thailand. As of 2010, 34 cases of human trichinellosis were documented in Korea, marking its first outbreak in 1997 [6]. The travel history of the patient to Southeast Asia likely constitutes the source of infection, aligning with the geographic distribution and epidemiological patterns of the disease.

The life cycle of a parasite includes two stages: the muscular (parenteral or systemic) phase and the GI phase. After the contaminated meat consumption, the GI phase begins with the release of the larvae into the stomach. They then migrate through the mucosa of the small intestine, maturing into adult worms within four to five days postinfection. Following copulation, female worms release newly formed larvae into the lymphatic system. During the muscular phase, the larvae leave the GI mucosa and disseminate throughout the body via the bloodstream. The invasion and prolonged residency of these larvae in the striated skeletal muscle cells lead to three significant cellular changes: the encapsulation of the larvae, the degradation of sarcomere myofibrils, and the creation of a vascular network surrounding the infected cells [7]. The incubation period can vary from one to four weeks.

The typical symptoms of trichinellosis include fever, periorbital edema, myalgia, and gastrointestinal manifestations such as vomiting, diarrhea, and abdominal pain. In severe cases, additional complications such as pneumonia, pleuritis, encephalitis, and eye disease have also been reported. Furthermore, cardiovascular problems can occur in 10-60% of individuals with trichinellosis, a common infectious disease. The majority of cardiac damage is observed during the invasive infective stage of the disease. The cardiovascular issues that were previously discussed include Takotsubo cardiomyopathy, pericarditis, myocarditis, and thromboembolism [8]. Nonetheless, trichinellosis rarely results in mortality. An international assessment of trichinellosis done by the International Commission on Trichinellosis between January 1995 and June 1997 revealed 20 deaths out of 10,030 cases [7]. In this case, the patient’s initial presentation with cardiogenic shock, given his history of a bicuspid aortic valve, was initially misdiagnosed as significant aortic stenosis. The coexistence of myopathy and eosinophilia suggested the potential of another underlying inflammatory condition, suggesting further investigation into other etiologies of hypereosinophilic syndrome or parasitic infection.

Eosinophilia is commonly observed at the time of a trichinellosis diagnosis, like in this case. Alternative differential diagnoses, including drug rash with eosinophilia and systemic symptoms (DRESS) syndrome, should always be considered in cases of significant eosinophilia; yet, the lack of a medication history in this patient made this particular diagnosis less likely. Elevated cardiac enzymes, including troponin and creatine kinase, are frequently noted in cardiac trichinellosis. Atrial fibrillation, right bundle branch block, first-degree atrioventricular block, sinus bradycardia or tachycardia, and supraventricular premature beats may all be seen on an ECG. Such patterns of elevated cardiac enzymes alongside eosinophilia have been consistently documented in previous case reports, highlighting the cardiac sequelae associated with trichinellosis. Specifically, a case of trichinellosis-induced myocarditis reported by Mohib et al. [9] identified an elevated Troponin T high sensitivity level at 785 ng/L (normal range: <14 ng/L) and an increased eosinophil count at 7,440/uL (normal range: <400/uL). This pattern of elevated cardiac enzymes and eosinophil counts has been consistently reported in previous case studies. Conversely, a case of *T. spiralis* pericarditis documented by Nkoke et al. [10] did not present with eosinophilia in the complete blood count, suggesting that eosinophilia might occasionally be absent in such parasitic infections. This indicates the importance of considering trichinellosis in the differential diagnosis, even in the absence of eosinophilia.

Although the patient was unable to undergo an endomyocardial biopsy and cardiac MRI due to an unstable condition at the time, treatment was initiated with a high-dose corticosteroid for presumed eosinophilic myocarditis. Despite not fulfilling the criteria for myocarditis due to the absence of histopathological evidence [11], the patient’s condition gradually improved with the high-dose corticosteroid and subsequent antihelminthic therapy. Eosinophilic myocarditis is a form of inflammatory cardiomyopathy characterized by the infiltration of eosinophils into myocardial tissue and can be associated with parasite infections, malignancies, or idiopathic hypereosinophilic syndrome [12]. The patient’s favorable response to corticosteroid therapy is consistent with the anticipated outcomes for eosinophilic myocarditis, suggesting that the initial diagnosis was likely accurate despite the diagnostic challenges encountered.

A muscle biopsy may provide a conclusive diagnosis of trichinellosis if it reveals the encysted larvae; however, not all patients will benefit from this technique. As a result, serologic testing might be useful for the diagnosis; the most often used test is the enzyme-linked immunosorbent assay, which has 99% sensitivity and 91-96% specificity [8]. Ultimately, the association between different clinical symptoms and pertinent test findings is what determines if an infection is diagnosed. Although endomyocardial biopsy is the gold standard for diagnosis, its applicability may be restricted in patients who are critical or who cannot endure the procedures, as in this case. Rather, trichinellosis was confirmed by a rectus femoris muscle biopsy, which emphasizes the need to take other biopsy sites.

Antihelmintics and glucocorticosteroids are among the medications used for treatment. Several antihelmintics, including albendazole and mebendazole, are well known for their effectiveness. Drug treatment is most effective when initiated within one week (the early stage) after an infection; however, diagnosing an infection before symptoms appear can be challenging. As a result, it is often recommended that patients begin medication four to six weeks after infection. To prevent immediate-type hypersensitivity reactions, azole is recommended along with glucocorticoid therapy. In this case, the combination of albendazole, ivermectin, and methylprednisolone resulted in the complete resolution of symptoms and normalization of cardiac biomarkers.

## Conclusions

The significance of including trichinellosis in the differential diagnosis of myocarditis is highlighted in this case. As seen in this patient with underlying bicuspid aortic valve disease, trichinellosis can lead to severe cardiac symptoms, although it is rare. Prompt diagnosis and appropriate treatment with glucocorticoids and anthelmintic medications are crucial for achieving favorable outcomes. Healthcare practitioners should be vigilant for parasitic infections in patients presenting with nonspecific cardiac symptoms, especially if there is a history of consuming raw or undercooked meat in an endemic area.
